# Case of Retroflexion of the Uterus, of Three Years’ Standing, Cured

**Published:** 1866-10

**Authors:** John H. Griscom

**Affiliations:** New York


*Case of Retroflexion of the Uterus, of Three Years' Standing, Cured.* By John H. Griscom, M. D., New York.

In No. 482, May 26, of the Reporter, are related two cases of uterine flexion, which were remarkable for their extensive and complicated sympathetic effects, one upon the pulmonary and nervous, the other upon the digestive systems; the latter especially notable for the severe dyspepsia and constipation caused by it. In each of those cases, the uterus was restored to its natural position, and all the sympathetic symptoms relieved by very simple means. Simpson's sound was used to reduce the flexion, and this being accomplished, a simple horseshoe pessary, aided by a continued recumbent posture for a few days, sufficed to retain the organs *in situ* long enough to enable the local tissues to become confirmed in tone, and preserve the organs in place.

Another case which lately came under my care, presented still another variety of sympathetic influences, and a combination of symptoms requiring quite a different course of treatment. The patient, a lady from a distant State, sought my counsel in June last, complaining of great distress in the pelvic region, and great prostration and disturbance of general health. Her age is 27, has been married nine years, and has borne three children, besides having had two miscarriages.

An examination per vaginam revealed a most decided retroflexion, the fundus pressing in upon the rectum to such a degree as to completely obliterate its cavity, although the uterus presented no evidence of enlargement. It was readily relievable from its position by the finger alone, so far as it could reach, which was of course insufficient to restore it fully to its normal position. Digitation produced considerable pain, in consequence of a marked tenderness of the vaginal membrane, a discouraging circumstance in appearance, as the use of a pessary of any kind requires a healthy condition of the parts, to enable it to be borne with comfort and efficiency. Observation with the speculum, however, revealed nothing more than a slight redness of the parts, unaccompanied with any thing like ulceration. There was also a decided relaxation and expansion of the posterior *cul de sac*, which, though favoring the use of a pessary, presented a prospect of such a general relaxation of the tissues as would be likely to delay a permanent restoration of the misplaced uterus.

Encouraged by previous successes to rely upon the efficacy of the horseshoe pessary, in combination with a continued recumbency of body, after replacing the uterus by the sound, this course was tried for three or four days, but the excessive heat of the weather rendering confinement to the bed almost unendurable, and the enlarged capacity of the vagina rendering the pessary less available than ordinary, it became necessary to abandon that method of treatment.

About a year previous to my acquaintance with the case, it had been treated with the stempessary, which, while it remained in its place, of course retained the uterus in proper position, but during menstruation, it became necessary to remove it, thus failing to accomplish the desideratum.

Having become acquainted a short time before with the instrument invented by Dr. Banning, and examined the variety of forms of his "balance," and their adaptability to different abnormal positions, I concluded to try it, and accordingly selected the one which seemed most applicable to the case in hand.

The first object in all cases of uterine flexion is, of course, the restoration of the organ to its natural attitude, and the maintenance of it therein to enable the relaxed and disordered tissues to recover their tone and contractility, so that when the instrument is removed, the uterus will preserve its natural position. No form of instrument can be relied upon for the *permanent and radical* cure of the disorder, but as the restoration of the position of the organ is the first and essential performance, the choice of the most available form of instrument for this purpose is a matter of much importance in each case.

In the case under consideration it was found necessary, after a brief trial of that part of the apparatus which lies within the vagina and supports the fundus in its natural position, to change its curve, so as better to adapt it to the relaxed parts, and make a more direct pressure upon the uterus itself. The substance of which the stem of the "balance" is made, (hard rubber,) enables this to be done with very little trouble, so that after a short trial the arrangement was well adapted to the circumstances of the case. Aided by the external supporter, to which the internal balance is attached, and by it kept steadily in place, my patient was enabled to exercise herself by walking and riding in a gentle manner, and thus materially aid in the restoration of her general health, which had been for a long time much depressed.

The final result has proved as satisfactory as could be desired. After about six weeks' continued use of the instrument, occasionally interrupted by the necessity of its temporary removal for the purpose of applying astringent and soothing injections, the uterus was found to be completely restored to its natural position, and maintained it completely for a fortnight, subsequent to the removal of the instrument, at which time the patient left for her home, with confidence in its continuance.

Injections of tannin in solution, when the parts were most relaxed, and applications of oxide of zinc, dissolved in glycerine, (ʒj. to the f. ℥j.,) by means of tampons of cotton, to relieve the inflammatory tendency, had the effect desired, while the internal administration of tonics and ferruginous preparations so far restored the patient's general health, as to enable her to take full exercise of body, without any fear of a return of the original trouble.—*Medical and Surgical Reporter.*

